# *Paramecium* Genetics, Genomics, and Evolution

**DOI:** 10.1146/annurev-genet-071819-104035

**Published:** 2023-11-27

**Authors:** Hongan Long, Parul Johri, Jean-Francois Gout, Jiahao Ni, Yue Hao, Timothy Licknack, Yaohai Wang, Jiao Pan, Berenice Jiménez-Marín, Michael Lynch

**Affiliations:** 1 Institute of Evolution and Marine Biodiversity, KLMME, Ocean University of China, Qingdao, Shandong Province, China; 2 Laboratory for Marine Biology and Biotechnology, Laoshan Laboratory, Qingdao, Shandong Province, China; 3 Department of Biology, University of North Carolina, Chapel Hill, North Carolina, USA; 4 Department of Biological Sciences, Mississippi State University, Starkville, Mississippi, USA; 5 Cancer and Cell Biology Division, Translational Genomics Research Institute, Phoenix, Arizona, USA; 6 Biodesign Center for Mechanisms of Evolution, Arizona State University, Tempe, Arizona, USA

**Keywords:** ciliates, evolutionary cell biology, gene duplication, genome evolution, *Paramecium*, population genomics

## Abstract

The ciliate genus *Paramecium* served as one of the first model systems in microbial eukaryotic genetics, contributing much to the early understanding of phenomena as diverse as genome rearrangement, cryptic speciation, cytoplasmic inheritance, and endosymbiosis, as well as more recently to the evolution of mating types, introns, and roles of small RNAs in DNA processing. Substantial progress has recently been made in the area of comparative and population genomics. *Paramecium* species combine some of the lowest known mutation rates with some of the largest known effective populations, along with likely very high recombination rates, thereby harboring a population-genetic environment that promotes an exceptionally efficient capacity for selection. As a consequence, the genomes are extraordinarily streamlined, with very small intergenic regions combined with small numbers of tiny introns. The subject of the bulk of *Paramecium* research, the ancient *Paramecium aurelia* species complex, is descended from two whole-genome duplication events that retain high degrees of synteny, thereby providing an exceptional platform for studying the fates of duplicate genes. Despite having a common ancestor dating to several hundred million years ago, the known descendant species are morphologically indistinguishable, raising significant questions about the common view that gene duplications lead to the origins of evolutionary novelties.

## 1. INTRODUCTION

**Figure 1:**
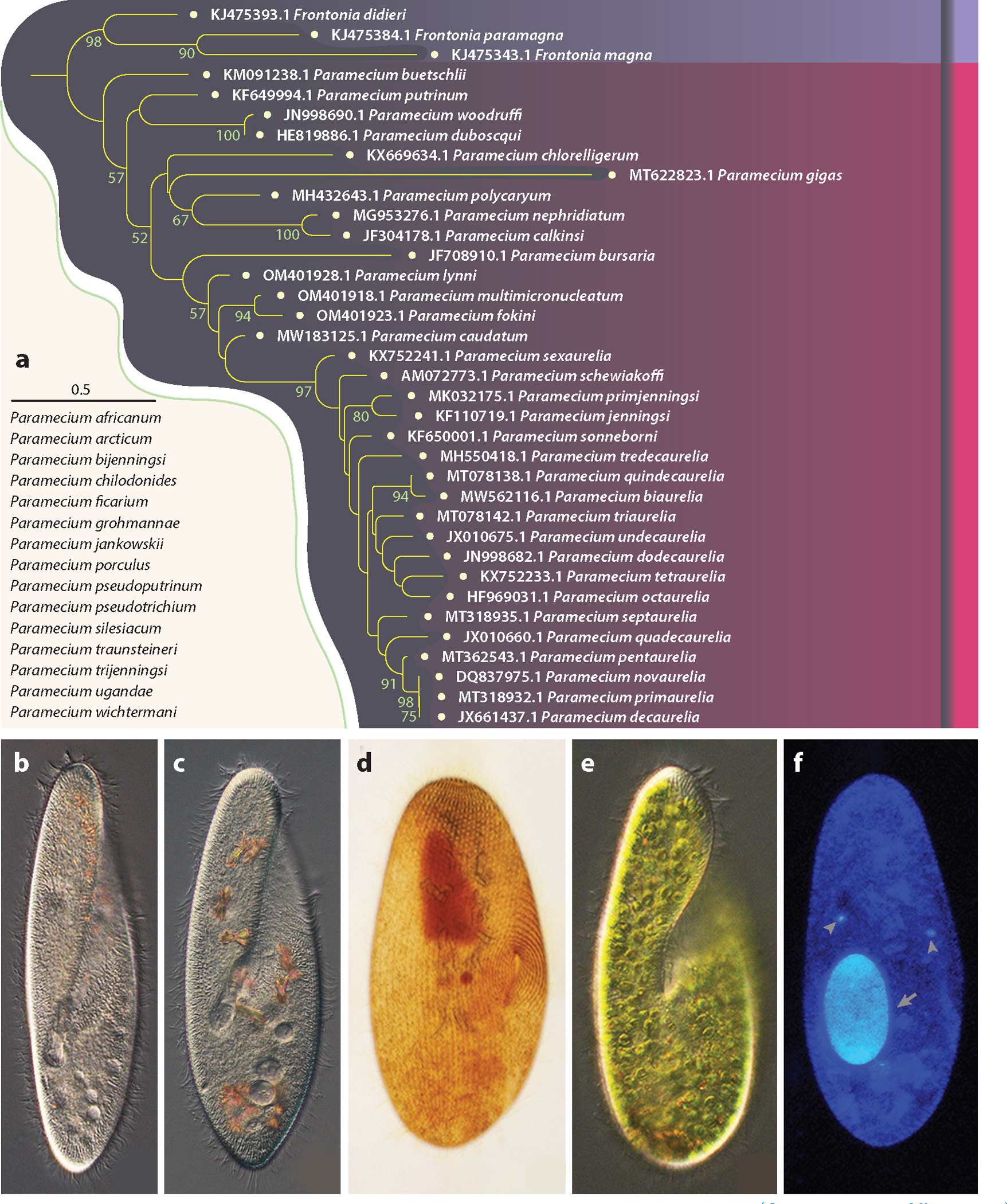
Phylogeny and morphology of *Paramecium* species. (*a*) A maximum-likelihood tree based on mitochondrial *coxI* sequences [National Center for Biotechnology Information (NCBI) GenBank Accession numbers are given as prefixes]. Species under the scale bar (0.5 substitutions per site) do not have available *coxI* sequences; nodes without numbers have bootstrap values <50. (*b*) *Paramecium tetraurelia*, (*c*) *Paramecium biaurelia*, (*d*) *Paramecium biaurelia* after ammoniacal silver carbonate staining, (*e*) *Paramecium bursaria*, and (*f*) *Paramecium biaurelia* after Hoechst 33342 staining.

*Paramecium*, a ciliate genus containing at least 48 species (Figure 1a), has fascinated scientists since the dawn of microscopy. Some *Paramecium* are morphologically indistinguishable, even in the infraciliature after silver staining—the gold standard of modern ciliate morphological taxonomy (Figure 1b–d)—and must be subjected to mating tests for proper assignment, whereas others are highly recognizable morphologically (Figure 1e). Most species are cosmopolitan with large enough cell sizes (lengths ≈ 50–300 μm) to be just visible to the naked eye (125). As with all other ciliates, each cell harbors two types of nuclei—a germline micronucleus and a somatic macronucleus (Figure 1f). The micronucleus divides mitotically but remains transcriptionally silent during vegetative growth and then undergoes meiosis during conjugation and autogamy. The diploid germline genome sequestered in the micronucleus, which contains 30–63 chromosomes in members of the *Paramecium aurelia* species complex (119), carries the complete genetic information for the progeny, as well as internally eliminated sequences (IESs) that do not exist in the macronucleus and are excised during the development of the new macronucleus via sexual processes. By contrast, the macronucleus contains a highly polyploid genome (with hundreds of copies of each chromosome, each with a substantial amount of DNA excised during maturation from the micronucleus) (87). The macronucleus is constantly transcriptionally active, divides amitotically, and is discarded and replaced with a new version developed from the micronucleus after mating (2, 32).

Numerous ciliatologists have made great contributions to *Paramecium* studies. Most notably, Herbert Spencer Jennings (118) and Tracy Morton Sonneborn (10) laid the foundation for modern *Paramecium* biology. Jennings greatly promoted the understanding of the life cycle and behavior of *Paramecium* and broke the ground for all subsequent protozoan genetics studies, contributing to the early emerging field of mathematical genetics and educating numerous people with his book *The Biological Basis of Human Nature*. Sonneborn, initially a graduate student and research assistant of Jennings, discovered the two mating types in the *P. aurelia* species complex; made *Paramecium* genetics technically feasible; and performed fundamental work on infectious heredity (via Kappa particles, which would later be found to be bacterial in nature) (100, 117), cortical inheritance, and aging and rejuvenation, among others. More recently, John R. Preer Jr., a graduate student of Sonneborn who also made major contributions to *Paramecium* biology, reflected on the current field, lamenting that despite its central role for decades as a model organism (11, 129), *Paramecium* had become close to an “endangered genetic species” (102, p. 222). The misconception that the idiosyncrasy of the *Paramecium* life cycle is irrelevant to mainstream genetics might have been a contributing factor here. Ironically, the presence of a silent germline nucleus and a disposable somatic nucleus provides a unicellular analog of the typical situation in multicellular eukaryotes.

Now well into the omics era of technology, few *Paramecium* genetics labs remain, although substantial discoveries are still being made. Here, we integrate these recent results with the historical legacy of studies in *Paramecium* genetics. We focus, in particular, on the genetics and evolutionary genomics of *Paramecium*, noting that recent systematic reviews have appeared on related topics, such as genome rearrangement and epigenetics during sexual processes (31, 42, 124) and evolutionary bioenergetics (81).

## 2. GENETICS

### 2.1. Model Species

Among *Paramecium* species, members of the *P. aurelia* species complex [Latin name first given by O.F. Müller (91)] have drawn the most attention. Sixteen sibling species have been reported (6, 101, 119, 129), all with cell lengths of 90–170 μm, and are morphologically indistinguishable but sexually incompatible and substantially divergent at the molecular level (43) (Figure 1a,b). Despite his observations from crossings, isozyme gel patterns, and other features, Sonneborn (10) was initially hesitant to assign Linnean Latin names to the members in the cryptic species complex, referring to them as syngens, with the only objective criterion for subsequent strain identification being through the use of a mating type panel. Fortunately, along with many other *Paramecium* species, the legacy of the mating panels for all 15 species that Sonneborn deposited in the 1940s [including *Paramecium sonneborni*, named after him by Aufderheide et al. (6)] is still available at the American Type Culture Collection. The type strain of recently reported *Paramecium quindecaurelia* is available at the Resource Centre Culture Collection of Microorganisms (RC CCM) of Saint Petersburg State University, Russia (101).

**Figure 2:**
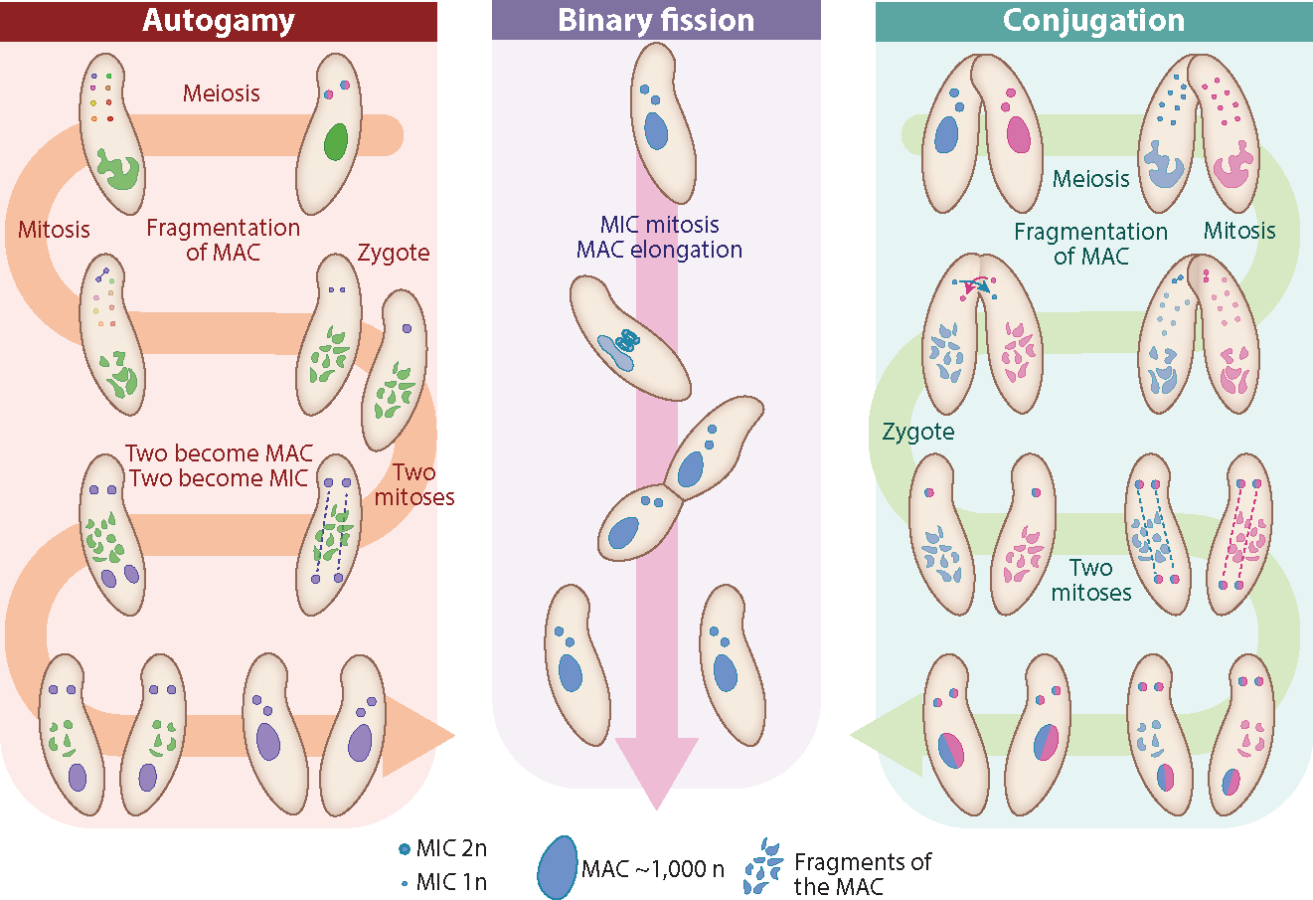
The life cycle of *Paramecium*. Note that there are two MICs in most *Paramecium aurelia* species; for simplicity, in this diagram, conjugation and binary fission start with homozygous nuclei. Abbreviations: MAC, macronucleus; MIC, micronucleus.

*P. aurelia* species reproduce asexually by fission but also undergo sexual processes involving conjugation (between two cells with different mating types) or autogamy (within one cell, progeny from autogamy are 100% whole-genome homozygous in both nuclei), both of which involve meiosis that is usually induced by starvation (Figure 2). During macronuclear development, sequences that are uniquely present in the micronucleus (IESs) are eliminated from macronuclear chromosomes. This genome rearrangement process involves the use of small scanning RNAs generated in the micronucleus and shuffled between the old macronucleus and the new macronucleus. The development of the new macronucleus is thus under epigenetic control. As recent reviews have sufficiently summarized this topic (31, 42, 124), we do not go into detail here. The essential point is that once eliminated from the macronucleus, a particular sequence will remain so after future episodes of sexual reproduction. If a micronuclear-specific sequence is injected into the macronucleus, it will be retained there following future sexual phases (93).

Other well-studied *Paramecium* models with a long scientific history include the *Paramecium bursaria* species complex, with at least five members (120). These species naturally stand out when observed with other microbes under microscopes, owing to their relatively large cell size and green color conferred by the endosymbiotic green algae *Chlorella* species, and thus have long been a model for studying algal symbiosis (23, 54, 64, 84) (Figure 1e). Many other endocytobionts, including bacteria and fungi (130), are found throughout the *Paramecium* genus, for example, in the extracellular cortex (19), cytoplasm, macronucleus, and micronucleus (103, 127). Other *Paramecium* with even larger sizes, such as *Paramecium caudatum* and *Paramecium multimicronucleatum* (170–310 μm in length), have served as model species for cell biology, genetics, and ecotoxicity and have recently been studied from the standpoint of genome evolution (60, 85, 112).

### 2.2. Mating Types

Contrary to the common conceptualization of sexual reproduction, where gametes from two parents fuse to form a zygote, conjugation in *Paramecium* consists of the reciprocal exchange of a haploid nucleus between two sexually reactive cells, without the production of a new zygote. Because there is no difference in gamete size to allow for the differentiation of sexes, the different sexes of *Paramecium* (and other unicellular organisms) are called mating types. As mentioned above, mating types in *P. aurelia* were discovered by Sonneborn (116), a discovery that led to the realization that *P. aurelia* is in fact a complex of multiple morphologically identical species, only identifiable through conjugation tests. These mating types were eventually named odd (O) and even (E) for each species. Early genetic studies revealed that three (unlinked) genes (*mtA*, *mtB*, and *mtC*) are necessary for the expression of the mating type E in *Paramecium tetraurelia* (17).

The first round of binary fission after mating produces two pairs of young cells (one from each exconjugant) called karyonides. The distribution pattern of mating types among these four karyonides reveals the mode of inheritance of the parental mating types (reviewed in 96). Surprisingly, the mode of mating type determination varies widely among species of the *P. aurelia* complex (96). In some species, mating type determination for the four karyonides is stochastic, with no correlation to the parental mating type (this is called a karyonidal pattern). Other species display a Mendelian pattern as expected if the mating type is encoded in the germline genome (this is called a synclinal pattern). In some species, the two pairs of karyonides each express the mating type of their cytoplasmic parent, suggesting a transmission mechanism independent of the germline DNA sequence. This cytoplasmic pattern implies that cells with a similar micronucleus but a different cytoplasm express different mating types. An obvious candidate for the production of different phenotypes from the same germline genotype in *Paramecium* is differential rearrangements in the macronucleus. Therefore, it has long been suspected that mating type in these species is determined by differential rearrangements of the macronucleus resulting in the expression of different genes between the two mating types.

In mating type determination in *P. tetraurelia*, the *mtA* gene encodes a transmembrane protein whose N-terminal region extends outside of the plasma membrane and most likely is involved in the agglutination between complementary cells. The differential retention of an IES at the 5′ end of the *mtA* gene is responsible for mating type determination (114). Excision of this IES results in a truncation of the first few coding nucleotides and of the promoter of *mtA*, preventing the expression of the *mtA* protein and resulting in cells of type O (114). More recent investigations have shown that the same mechanism operates in the closely related *Paramecium octaurelia*, *Paramecium decaurelia*, and *Paramecium dodecaurelia* (110). This study also revealed a diversity of molecular mechanisms responsible for mating type determination in other members of the *P. aurelia* group. In *Paramecium sexaurelia*, the mating type is also determined by the differential retention of an IES in *mtA*, but this IES is located in the 3′ end of the *mtA* gene. *Paramecium biaurelia* and *Paramecium septaurelia* both rely on differential excision of two nonhomologous IESs inside the *mtB* gene, of which expression is necessary for *mtA* expression. Finally, the Mendelian inheritance pattern of mating types in *Paramecium tredecaurelia* was shown to be caused by a mutation in the 5′ end of its *mtA* gene, resulting in two alleles: a dominant one expressing mating type E and a recessive allele determining mating type O (91).

The rapid evolution of so many different mechanisms of mating type determination in a single cryptic species complex makes the *P. aurelia* system a compelling model for studying the evolution of sex and all the questions related to self-incompatibility in eukaryotes. Likely, many additional mechanisms await discovery in other *Paramecium* species, but how such transitions arise and become established evolutionarily remains unclear. Autogamy, which is unique to the *P. aurelia* complex, may facilitate such evolution by leading to the rapid local fixation of new types.

### 2.3. Intron Splicing

While the many oddities of *Paramecium* and ciliates in general might appear as mere curiosities, they can reveal interesting patterns that had remained elusive in other model organisms. This is the case for selection against intron translation, which was first discovered in *Paramecium* and turned out to be a general eukaryotic phenomenon. *Paramecium* has extremely short introns, with more than 95% of them being between 20 and 34 nucleotides long (33, 106, 131). This challenged our understanding of the requirements for splicing and also yielded insights into the consequences of missplicing (56). The size distribution of *Paramecium* introns exhibits an obvious deficit of introns whose lengths are a multiple of three. This pattern was shown to result from selection to mitigate the consequences of intron retention, defined as the failure of the spliceosome to remove an intron before translation. Splicing is an imperfect mechanism, and intron retention is a common phenomenon (40). The consequences of these splicing failures depend greatly on the capacity of surveillance mechanisms to detect aberrant transcripts before they undergo translation. Introns whose length is not a multiple of three cause a frameshift when retained, resulting in the appearance of downstream premature termination codons (PTCs) that trigger nonsense-mediated RNA decay (NMD). Degradation of such faulty messenger RNAs (mRNAs) prevents the production of aberrant proteins, reducing the energetic costs, and preventing the production of misfolded proteins with potentially harmful functions. The retention of introns whose size is a multiple of three can still trigger NMD if they contain at least one in-frame stop codon. However, the retention of translatable introns (i.e., introns whose size is a multiple of three and that do not contain any in-frame stop codons) cannot be detected by NMD, leading to the translation of these potentially harmful transcripts. Therefore, it is expected that selection should result in a depletion of such translatable introns. Because introns are so short in *Paramecium*, and their genetic code contains only one stop codon, the probability that an intron encodes an in-frame stop codon just by chance is extremely low. Therefore, selection against translatable introns operated primarily on the size of introns, leading to a depletion in introns whose size is a multiple of three. Upon closer inspection, selection against translatable introns was found to be universal for intron-rich eukaryotes, including humans. This pattern had been overlooked in the eukaryotes with long introns, most of which carry at least one in-frame stop codon by chance. Studies that focused on the subset of introns that do not contain any in-frame stop codon in other eukaryotes (*Arabidopsis thaliana*, *Homo sapiens*, *Caenorhabditis elegans*, *Drosophila melanogaster*, and *Schizosaccharomyces pombe*) found that they produced a pattern similar to that of *Paramecium*, revealing that the universal threat caused by translatable introns in *Paramecium* helped shed light on the selective pressures imposed on eukaryotic genomes by the imperfections of the splicing machinery (56, 109).

The *Paramecium* genome also contains evidence for selective pressures against sequences that could result in aberrant splicing of functional exonic sequences (i.e., exonic sequences that match the GTA/TAG canonical splice sites). It also revealed that splicing of exonic sequences is a major mode of new intron gain (107).

### 2.4. Genetic Tools

Beyond the screening of naturally occurring and lab-induced mutant strains (68), molecular-genetic manipulations in *Paramecium* are mostly transient in nature. The two most popular methods are the direct injection of foreign DNA into the macronucleus (DNA microinjection) (13) or the temporary knocking down of target genes through RNA interference (RNAi) (41). DNA microinjection remains the gold standard for introducing transgenes and assaying protein localization and expression in any *Paramecium* species. DNA constructs—either linear or circular—are cloned into an expression vector that can then be manually injected into the *Paramecium* macronucleus, where its gene products are expressed with the host transcriptional/translational machinery. This has provided a powerful means of rescuing mutant phenotypes via the injection of wild-type sequences. Additionally, constructs can be designed to fuse proteins of interest with a codon-optimized green fluorescent protein (GFP)—a protein sequence from the jellyfish *Aequorea victoria* (51). GFP fusion proteins solved a key issue in *Paramecium* cell biology in which antibodies raised against a protein of interest often reacted with multiple paralogs that became indistinguishable when imaged. This was the case for the first application of this technology, *P. tetraurelia* homologs of the sarco(endo)plasmic calcium ATPase genes *ptSERCA1* and *ptSERCA2* (52, 63), which colocalize to the endoplasmic reticulum and alveolar sacs (53).

Macronuclear microinjection has several limitations. First, the transformation is transient in nature, as the DNA construct is lost following the next phase of sex, when the macronucleus is regenerated. Second, the method is very low throughput and can only be performed on one cell at a time. Thus, a high degree of technical experience is needed to perform routine transformation of candidate genes. Transforming large cultures of *Paramecium* remains difficult despite being routine in the related ciliate *Tetrahymena thermophila* (22). Despite some success at applying electroporation (15), biolistics (39, 66), and arginine-rich particles (25), high-throughput transformation of batch cultures is very inefficient and rarely done in practice.

RNAi knockdown provides the only means of altering gene function in batch cultures, and this is done simply through feeding (41), in a process similar to the one originally performed in *C. elegans* (37). Double-stranded RNA (dsRNA) is designed to target a gene of interest, cloned into a construct to be expressed in *Escherichia coli*, and then *E. coli* is fed to *Paramecia* in lieu of their original food. When ingested, bacterial dsRNA is processed by a similar protein machinery as that in *C. elegans*, for example, Dicer (69, 83). The knockdown efficiency of the target gene can be accessed with northern blots or reverse transcription–quantitative polymerase chain reaction (RT-qPCR). However, there is no current way to modulate the level of knockdown (i.e., strongly or weakly), and some genes are resistant to knockdown for either biological or technical reasons. Nonetheless, in a number of applications, combining RNAi with RNA sequencing or phenotyping assays has proven a powerful way to study gene function, even in highly similar paralogs that can be targeted separately or simultaneously [i.e., double-knockdowns (16)]. This has been particularly effective in elucidating the mechanisms by which RNA-mediated genome rearrangement is achieved (1, 27, 89).

## 3. EVOLUTIONARY GENOMICS

### 3.1. Genomic Resources

Ever since the first report on the high-quality whole macronuclear genome of *Paramecium*, *P. tetraurelia* d4–2, was published (7), *Paramecium* has been the ciliate genus with the most abundant genomic resources, including the nuclear and the linear mitochondrial genomes, most of which can be easily accessed through ParameciumDB (4, 61) or the National Center for Biotechnology Information (NCBI) Genome and Assembly databases (Supplemental Tables 1 and 2) (7, 43, 85). The macronuclear genome is highly streamlined and AT rich, with gene numbers almost twice as high as those of humans for the *P. aurelia* species complex, making *Paramecium* one of the most gene-rich microbial eukaryotes known (Supplemental Tables 1 and 2). Many local syntenies are also retained in *Paramecium* genomes (85). Explorations of the micronuclear genomes are less extensive but still reveal numerous insights, including the excision mechanisms and diversification of IESs (3, 5, 112). Unlike many other ciliates (62, 104), *Paramecium* does not have gene scrambling; that is, the within-gene order of exons in the macronucleus is the same as in the micronucleus. Given the established methods for macronuclear isolation and flow cytometry–based micronucleus isolation (47, 115) and the emergence of long-read sequencing technologies, many more high-quality *Paramecium* genome assemblies are expected in the near future.

**Figure 3:**
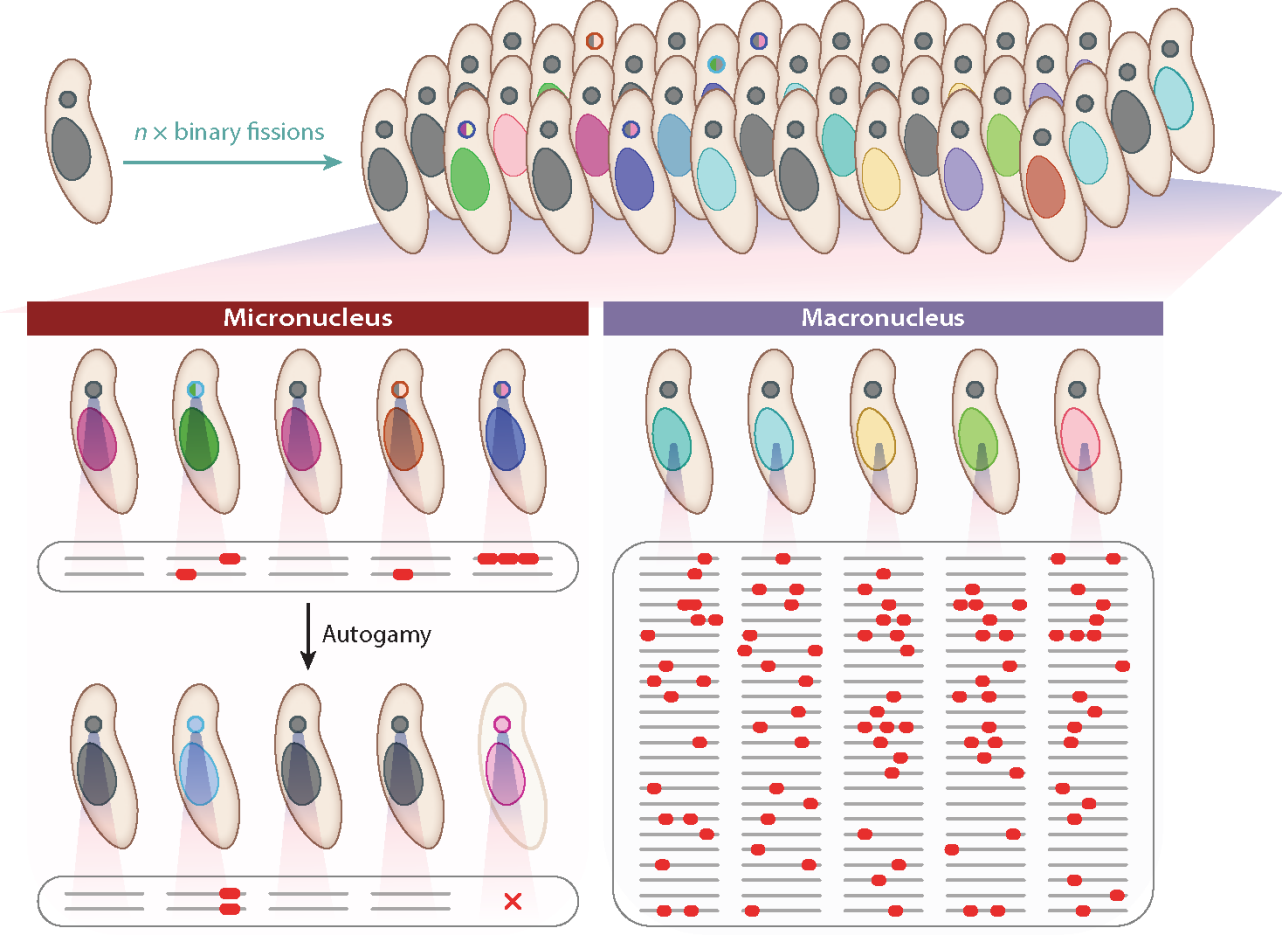
Illustration of micronuclear versus macronuclear mutations. In a layout similar to that in multicellular animals, ciliates (in a single cell) harbor a transcriptionally silent germline nucleus (*small dots*) and a transcriptionally active macronucleus (*large blobs*), which is disposed of and replaced following meiosis via autogamy or conjugation. (*Left*) In the micronucleus, mutations accumulate neutrally during vegetative growth because transcription is confined to the macronucleus. However, these are then exposed after autogamy/conjugation and the construction of a new macronucleus from prior micronuclear material. Ostensibly, to reduce the high mutation load after extended periods of asexual reproduction, *Paramecium* species have evolved very low micronuclear mutation rates. (*Right*) This panel illustrates the possibility that the fitness effects of mutations accumulating in the macronucleus may be very small as a consequence of the masking effects resulting from the polyploid nature of the macronuclear genome. Not shown, however, is the potential drift of numbers of macronuclear copies of individual chromosomes and resultant stoichiometric imbalance.

Numbers of protein-coding genes vary greatly among *Paramecium* congeners, with the highest being 49,951 in *P. sonneborni* strain 30995 and the lowest 8,713 in *P. caudatum* strain 43c3d. Likewise, the genome sizes fit nicely to the levels of whole-genome duplications (WGDs) (Supplemental Tables 1 and 2; see also Section 3.4). By contrast, gene structures are highly conserved among different species. For example, 78–85% of genes contain introns, the lengths of these genes range from 1,357 to 1,482 bp with introns (1,272 to 1,412 bp without), and exons average 393 to 418 bp in length, with about 2 introns per gene. Consistent with the tiny introns of most ciliates, *Paramecia* have mean intron lengths of 23 to 28 bp. The intergenic regions are also short (60, figure 3), and untranslated regions (UTRs) are abundant (Supplemental Tables 1 and 2); that is, there are low levels of junk DNA, again demonstrating the high streamlining of the macronuclear genome.

### 3.2. The Population-Genetic Environment

*Paramecium* species are among the few microbial eukaryotes for which mutation rate estimates have been obtained, with the life cycle in members of the *P. aurelia* complex being particularly conducive to such measurement. As noted above, during vegetative growth of *Paramecium*, the micronucleus is transcriptionally inert, and as a consequence, all germline mutations accumulate in a neutral fashion. Mutation accumulation can then be carried out in replicate lines, maintained by single-progeny descent for hundreds to thousands of cell divisions. Taking such lines of *P. biaurelia*, *P. sexaurelia*, and *P. tetraurelia* through autogamy, the germline mutations sequestered within individual lines have been revealed in their homozygous states by whole-genome sequencing of macronuclear DNA, with normalization by the number of cell divisions and nucleotide sites assayed leading to the mutation rate per nucleotide site (70, 123).

Unicellular eukaryotes are known to have low mutation rates per nucleotide site per cell division, with the mutation rate 7.61 × 10^−12^ of the model ciliate *T. thermophila* being the lowest ever recorded (72, 73, 77). The base-pair substitution mutation rates of the *P. aurelia* species complex range from 1.94 to 2.44 × 10^−11^ per nucleotide site per cell division, with limited variation among species (70, 123). However, because of the limited number of mutations accumulated due to the low rates, the mutation spectrum (i.e., the frequency distribution of different mutation categories) of *Paramecium* has not been fully resolved. The limited number of base-pair substitution mutations in *P. biaurelia*, *P. sexaurelia*, and *P. tetraurelia* suggests a strong mutational bias with the rate of G/C to A/T mutation being 12.5 to 25 times that in the reciprocal direction (70). Such A/T mutation bias is typical in most organisms (29, 71, 82) but is in sharp contrast to the mitochondrial mutations, which are mostly biased in the G/C direction (61). The expected G/C content under mutation-drift equilibrium (3–7%) is much lower than that observed (14–24% for the fourfold degenerate sites), implying selection and/or gene conversion in favor of G/C content in opposition to the prevailing A/T mutation bias in most organisms (70, 72).

The mutational features reported in *Paramecium* are limited to those accumulated in the micronucleus, and no exploration has been conducted on the process in the macronucleus. According to the drift-barrier hypothesis (75, 76), because most mutations with fitness effects are deleterious, purifying selection is expected to push down the genomic mutation rate to the level at which the selective advantage from any further drop in the mutation rate is equal to the power of genetic drift. One potential reason for the particularly low mutation rates in ciliates relates to the nature of the sexual cycle. Because germline mutations remain transcriptionally silent during vegetative growth, they cannot be detected by natural selection until they are revealed at the macronuclear level following the next bout of sex. Because selection on the mutation rate operates on the time scale of sexual generations, it can be anticipated that the mutation rate per asexual division may be exceptionally low to maintain a reasonable overall rate per sexual cycle (123), just as the rate per human germline cell division is kept quite low despite the relatively high overall mutation rate per human generation (75) (Figure 3).

By contrast, the fitness effects of most accumulated macronuclear mutations may be diluted by other nonmutated alleles (assuming incomplete dominance) in the highly polyploid and disposable macronucleus. If this is the case, the strength of selection to lower the mutation rate in macronuclear DNA may be exceptionally low. We thus predict that macronuclear mutation rates will be higher than those in the micronucleus, analogous to the situation where there are much higher mutation rates in the somatic cells relative to germline cells of most eukaryotes (18, 75) (Figure 3).

Although the germline and somatic genomes are sequestered in the micronucleus and the macronucleus, respectively, the germ-soma development upon conjugation or autogamy introduces interactions that complicate mutagenesis mechanisms. For example, IESs are usually eliminated during the development of the new macronucleus. Occasionally, however, some IESs are retained in a small fraction of the alleles of a given locus in the macronucleus, especially under nonoptimal growth conditions, thereby introducing a unique structural form of somatic mutation (20, 126). Although the fitness effects of mutations are also critical to evolution, for ciliates, this problem has only been explored in *T. thermophila*, which has a mean fitness effect of 0.11, that is, an average 11% fitness drop per mutation, similar to what has been found in other organisms (35, 128), and thus this is a subject worthy of more research in the future (74).

Finally, we note that the dual nuclear environment is likely not the only factor associated with the evolution of low ciliate mutation rates. The degree to which replication fidelity can be improved by selection is a function of the magnitude of random genetic drift, which is inversely related to the effective population size (*N*_*e*_) (76). A common way to estimate *N*_*e*_ derives from the expectation that, under the assumption of neutrality, standing nucleotide diversity at silent sites has an expected value of *4N*_*e*_*u*, where *u* is equal to the base-substitution mutation rate per nucleotide site (128). Using the abovementioned mutation rate estimates to factor out 4*u*, observed levels of silent-site variation (21, 60, 70, 123) lead to the conclusion that for members of the *P. aurelia* complex, *N*_*e*_ is on the order of 2 × 10^7^ to 3 × 10^8^, whereas that of *T. thermophila* is ~10^8^ (70, 73). These estimates indicate that *N*_*e*_ in ciliates is higher than in any other unicellular eukaryote for which there are estimates, which further implies an unusually high efficiency of selection.

### 3.3. Population Genomics

Population-genetic analyses present a unique and approachable and in some cases the only possible avenue to learning about the biology, life history characteristics, and natural demography of unicellular species such as *Paramecium*. This is because population-genetic approaches can use sequence variation data within individuals to yield insights about historical processes operating on a natural population. While detailed work by Sonneborn (119) in *Paramecium* clearly demarcated well-defined species, much less is known about evolutionary processes contributing to molecular genetic diversity within and among *Paramecium* species. A number of the *Paramecium* species are globally distributed (21, 46, 105) and concordantly exhibit some of the highest levels of synonymous nucleotide diversity among eukaryotic species (8, 57, 60). As species identification in *Paramecium* has been experimentally determined by performing a large number of mating experiments (119), it is unlikely that these high values of genome-wide diversity are due to misclassification of species, as is further confirmed by clear monophyletic grouping of strains (60) using whole-genome sequencing data for both the macronuclei and the mitochondria. Such high values of nucleotide diversity in *Paramecium* populations, combined with very low mutation rates (70, 123), imply very large long-term effective population sizes, as noted above.

Analyses of population structure suggest that there is no clear pattern of spatial structure in *Paramecium* species (60), including no observed isolation by distance, which may contribute to the maintenance of a large local *N*_*e*_. Such a scenario is consistent with the “everything is everywhere” hypothesis that postulates that most microorganisms exist as large panmictic populations (36). However, one reason for skepticism here is that *Paramecium* species thrive only in freshwater and are not known to form cysts that would likely be necessary for long-distance transport such as cross-continental migration (119). More detailed characterization of population structure at smaller spatial scales (as compared to the current status of mostly single isolates per population) and estimation of migration rates (from patterns of shared nucleotide variation) should help distinguish between alternative population–structure scenarios.

Building a more accurate understanding of the spatial structure, migration rates, and fluctuations in sizes of individual populations/demes can be done with population-genetic methods that perform demographic inference, for example, *dadi* (48), *fastsimcoal* (34), and approximate Bayesian computation (12). Such methods identify the models that best explain the present allele frequency distributions or/and linkage disequilibrium (LD) patterns of neutral alleles across multiple populations. While extremely useful, accurately inferring the complex history of *Paramecium* populations may remain challenging for multiple reasons. First, selection is pervasive across their compact genomes (60); thus, both direct and indirect effects of selection will bias the inference of population size changes and will most likely require approaches that perform a joint inference of demography and selection (58). Importantly, while most traditional approaches that assume neutrality of synonymous sites will be biased if this assumption is not met, joint inference methods can account for selection acting on synonymous sites. Mutation accumulation studies that quantify the AT/GC bias, as done in Reference 72, can provide insights into whether synonymous sites are neutrally evolving in *Paramecium* species. Second, it has been suggested that human activities may have contributed to recent migration events between populations that are spatially quite distant from each other (38). This would result in the need to test the fit of many more evolutionary scenarios (for instance, considering scenarios where a pulse of migration may have occurred between all continents), increasing the search space for complex demographic models even further. Third, it is possible that unicellular species that live in freshwater have naturally complicated life histories, as small lakes and ponds can dry and freeze almost completely during summers and winters, respectively. In particular, it is possible for *Paramecium* populations to follow an extinction–recolonization model, where the relative rates of colonization from permanent sources (such as large lakes) and extinction can result in either very high or quite low differentiation between subpopulations (98). Such a model could explain the lack of clear geographical structure present in *Paramecium* species.

Accurate characterization of the life history and demographic processes in *Paramecium* species is important not only to further understand the natural ecology of the species but especially to build an appropriate null model for the inference of parameters and candidates of selection (59). A recent study employed population-genetic approaches and found that most new mutations at nonsynonymous sites in the *P. aurelia* species are strongly deleterious (with 2*N*_*e*_*s* < −100), where *s* is the selective disadvantage of the homozygote (59). Consistently, previous work using genome-wide nucleotide diversity values at different site types has yielded evidence of strong and pervasive purifying selection across the genomes (60), suggesting the presence of conserved regulatory regions near exons (60). Such a pattern is very much expected, as functionally important regions are likely to be enriched in the streamlined macronuclear genomes of *Paramecium*. Interestingly, fourfold degenerate sites were found to be the most neutrally evolving in these genomes, suggesting that such sites should be preferably used when performing inference of population-genetic parameters in *Paramecium* populations.

While strong purifying selection is evident in the nuclear genomes, the efficacy of purifying selection was found to be similar or even stronger in the mitochondrial genomes of *P. aurelia* species (61). This is in stark contrast to most multicellular species where mitochondria are expected to have much lower effective population sizes than the nucleus and thus are expected to accumulate more deleterious mutations than nuclear genomes (78, 92). In *P. aurelia* species, although mitochondrial genomes are usually uniparentally inherited and thus have a lower *N*_*e*_ than the nucleus, the incidence of autogamy can lead to a decrease in the nuclear *N*_*e*_, leading to potentially similar effective population sizes of the nuclear and organelle genomes. In *P. tetraurelia*, we can indeed infer a similar magnitude. The ratio of nuclear [*N*_*e*(*nuc*)_] to mitochondrial [*N*_*e*(*mit*)_] effective population sizes can be determined from *π*_*mit*_/*π*_*nuc*_/*d*_*mit*_/*d*_*nuc*_, where *π* is the expected heterozygosity and *d* is the rate of divergence at neutral sites (61, 82), leading to ratios in the range of 0.94 to 1.88 and suggesting that the effective population sizes of mitochondrial genomes are also in the range of ~10^7^ to 10^8^. Similar analyses in other *Paramecium* species (including those that do not undergo autogamy) would help develop a general hypothesis about the effectiveness of selection acting in the mitochondrial genomes of unicellular species.

Finally, future work is required to obtain average rates of recombination and the landscape of recombination across the genome in the *Paramecium* species. While a large amount of work in multicellular organisms has uncovered strong peaks of recombination hot spots in primate genomes and relatively more constant rates in other species such as *Drosophila* (121), almost nothing is known about the variance in recombination rates in unicellular species aside from a handful of species (121). Johri et al. (61) used population-genetic data in the mitochondrial genomes of *P. aurelia* species to look at the rate of decay of LD and found no evidence of recombination. This observation is highly consistent with previous experimental work in *Paramecium* that found no exchange of cytoplasm between parent cells during conjugation (65, 88). By contrast, the rate of recombination in the nuclear genomes can be anticipated to be quite large, as *Paramecium* species have large numbers of chromosomes (~100) but small genomes. With average chromosome sizes less than 10^6^ bp and an average of one crossover per chromosome for every conjugation (82), the average crossover rate per conjugation will likely be higher than 10^−6^ per site/conjugation. Thus, patterns of decay of LD in the nuclear genomes could provide insights into when nonindependent evolution at different sites became important. However, it should be noted that obtaining the landscape of recombination from population-genetic data could be challenging, as most such approaches assume demographic equilibrium and demographic history could bias the inferences about recombination (26).

### 3.4. Whole-Genome Duplication

The genome of *P. tetraurelia* revealed that the lineages in the *P. aurelia* complex have undergone at least three rounds of WGDs (here referred to as the old WGD, the intermediate WGD, and the recent WGD), with the old WGD occurring before the divergence of *Paramecium* from *Tetrahymena*, and the recent WGD happening before the emergence of the *P. aurelia* species complex (7). Later sequencing of the *P. caudatum*, *P. sexaurelia*, and *P. biaurelia* genomes confirmed that both the intermediate and the recent WGD were shared only within the *P. aurelia* species complex (85, 86). In newly formed polyploids, whole-genome doubling can cause genomic instability by disrupting chromosome segregation during meiosis (97). However, in *Paramecium*, such immediate deleterious effects may be mitigated by asexuality and autogamy, which enables single lineages of *P. aurelia* individuals to become established and sustained over long periods. Thus, it is possible that the *Paramecium* lineage is more permissive to WGDs, with polyploidy events being nearly neutral or slightly advantageous when they occur (85).

It was believed that the recent WGD in *Paramecium* involved autopolyploidy because of the limited unbalanced gene loss detected (7). However, one study showed that the recent *Paramecium* WGD could in fact be an allopolyploidy event, with clear statistical evidence supporting biased fractionation (49). Originally, the recent WGD was thought to be quite young, about 20 million years old, because the gene orders in these *P. aurelia* genomes are highly conserved, with only a few translocations and segmental inversions (7, 108, 123). But after correctly accounting for the widespread gene conversion between WGD duplicates in the *Paramecium* genomes, the recent WGD event was dated to about 320 million years ago (MYA) (86), using the extremely low mutation rate in *Paramecium* as a chronometer (70, 123). The intermediate WGD was estimated to be about 1.5 billion years old (85). However, molecular dating calibrated with Proterozoic protist fossils suggests that the divergence time between *Tetrahymena* and *Paramecium* was around 579.5 to 640 MYA (67, 99, 122). Thus, the margin of uncertainty is large, at least for the date estimates of the intermediate WGD, and the mutation rate–derived WGD date estimates may be viewed as the lower bounds of the timing of actual occurrences.

Regardless of the *Paramecium* WGD age estimation, the percentage of retained duplicates after the recent WGD is very high for *Paramecium* when compared to the gene retention rate in other postpolyploidy lineages in example species (43, 49, 108). The present-day genome of *P. tetraurelia* is densely packed with about 40,000 genes (7). The unbalanced gene loss and retention pattern after polyploidy is partly shaped by the selective constraints from gene dosage balance maintenance (14, 24). Indeed, Gout et al. (44) and Gout & Lynch (45) found that members of paralogous pairs that survive after polyploidy tend to be those that are highly expressed in *Paramecium*. A recent population-genomics study inferred the ongoing postpolyploidy gene loss process by identifying segregating loss-of-function mutations in gene duplicates in populations of *P. aurelia* species. During the process of loss, mildly deleterious mutations appear to gradually accumulate in coding or regulatory regions of one of the retained duplicates, causing expression or functional decline and eventually nonfunctionalization of the partially incapacitated copy (59). Together, these observations imply an important role of dosage constraints in postpolyploidy gene retention (50).

The recent WGD event is believed to be directly related to the explosive species radiation that formed the cryptic *P. aurelia* species complex, which consists of at least 16 sibling species (86, 119). Unlike multicellular lifeforms such as flowering plants (95) and vertebrates (28, 55, 94), in which polyploidy events putatively gave rise to morphological diversity and evolutionary novelties, despite the two rounds of WGDs, the *P. aurelia* species remain morphologically and ecologically indistinguishable (119). One of the mechanisms causing speciation is reciprocal gene loss (RGL), or the divergent resolution of duplicated genes (7, 79, 80, 113). RGL is a special case of the Bateson–Dobzhansky–Müller model of genomic incompatibility (9, 30, 90). After WGD, alternative copy members of duplicates can be independently lost in different subpopulations, subsequently resulting in hybridization incompatibility because F1 hybrids are presence/absence heterozygotes, producing null alleles in 50% of gametes. As RGLs continue to accumulate after WGD, nested speciation events can occur, leading to the origin of a species radiation, driven entirely by degenerative mutations (111). McGrath et al. (85) detected ongoing RGL of the intermediate WGD-derived orthologs, suggesting that ancient WGD is still helping to reinforce the reproductive isolation within the species complex.

## 4. CONCLUSIONS AND FUTURE DIRECTIONS

Although *Paramecium* has played a central role in the field of eukaryotic cell biology, over the past decade, it has also emerged as a model system for studies in population genetics and evolutionary genomics. The exceptionally streamlined and syntenically stable macronuclear genome, combined with the deep and well-characterized phylogeny of the genus, provides a powerful platform for evaluating the mode of evolutionary change at orthologous loci across vast evolutionary distances. *Paramecium* introns are among the smallest ever observed, and the macronuclear genome is nearly completely devoid of mobile genetic elements. Despite these simplified features, *Paramecium* genomes are well-endowed in terms of gene numbers. Most notably, the *P. aurelia* complex comprises a model system for studying the consequences of WGD, particularly given the extraordinary degree of morphological stasis despite substantial postduplication speciation over a span of hundreds of millions of years.

Given the joint presence of the transcriptionally silent germline micronucleus and the transcriptionally active somatic macronucleus, *Paramecium* and other ciliates provide a compelling contrast to studies of genomic and morphological evolution in multicellular animals, which also harbor a sequestered germline. The possibility that the substantial differences in the tempo and mode of evolution in these two systems are a consequence of a radical shift in the population-genetic environment cannot be ruled out. Mutation rates in the genus *Paramecium* are among the lowest observed in any organism, nearly an order of magnitude lower than rates in most prokaryotes, and effective population sizes are several orders of magnitude larger than those observed in metazoans.

Looking to the future, there is still a substantial need for work in the field of population genetics and genomics. To date, most within-species comparative work has relied on the characterization of single isolates from different populations, so the extent to which population subdivision versus global panmixia exists remains unclear. Given that there are no known mechanisms of desiccation resistance, how species have attained their global distributions remains a mystery. Further work is needed on the evolution of mating types, given the substantial divergence in mechanisms already known, as well as the presence of a high degree of cryptic speciation in numerous lineages. Moreover, almost nothing is known about the rate and patterning of recombination (crossing-over and gene conversion) within chromosomes. Compared to other eukaryotic cells of similar size, ciliates have some of the most rapid rates of cell division known, so deciphering the molecular mechanisms responsible for the extraordinarily low mutation rate in the germline nucleus could yield valuable insight into the evolutionary limits to replication fidelity.

With a deeper understanding of the evolutionary genetics and genomics now in place, and a substantial amount of prior cellular work to draw upon, the time is ripe for establishing *Paramecium* as a key model system in the emerging field of evolutionary cell biology. For example, with intergenic regions generally being only a few dozen base pairs in length, *Paramecium* harbors enormous potential for deciphering the evolutionary features of the regulatory vocabulary and underlying mechanisms governing gene expression levels. Macronuclear injection of engineered constructs (as well as RNAi) provides powerful ways to study the subcellular locations and functions of the products of single genes, but such genetic manipulations are transient. Of course, conventional crosses are possible, and members of the *P. aurelia* complex enable the rapid production of completely homozygous lines via single generations of autogamy. However, the major challenge to the full realization of the potential of *Paramecium* to yield the type of genome-wide understanding of cell biological features that has been obtained in other model systems is the need for methods for the stable integration of genetic modifications into the germline nucleus.

## Supplementary Material

ge57_lynch_suppltables1-2
