# Supplementary material for: Paramecium Genetics, Genomics, and Evolution
Source: Annu Rev Genet. Author manuscript; Available in PMC 2024 Aug 20. (PMC11334263; doi:10.1146/annurev-genet-071819-104035)
Supplement: ge57_lynch_suppltables1-2 [file NIHMS2015962-supplement-ge57_lynch_suppltables1-2.pdf]

Supplemental Material: Annu. Rev. Genet. 2023. 57:391-410
https://doi.org/10.1146/annurev-genet-071819-104035
Paramecium Genetics, Genomics, and Evolution
Long, Johri, Gout, Ni, Hao, Licknack, Wang, Pan, Jiménez-Marín, and Lynch

Supplemental Table 1 Genomic features of the *P. aurelia* species complex

A	B	C	D	E	F	G	H	I	J	K	L	M	N	O	P	Q
P. biaurelia	V1–4	76.98	25.78	a	40,261	1,427	81	31	40	407	2.35	26	1,347	21	30	459
P. decaurelia	223	71.91	27.32	a	40,810	1,373	79	38	36	393	2.34	26	1,292	23	33	347
P. dodecaurelia	274	71.63	27.33	a	41,085	1,384	78	43	43	399	2.32	26	1,298	27	36	335
P. jenningsi	M	65.35	23.17	a	37,098	1,357	78	43	37	397	2.27	26	1,272	28	37	380
P. novaurelia	TE	64.79	22.60	a	35,534	1,394	81	39	27	393	2.39	26	1,317	20	30	419
P. octaurelia	138	86.44	27.78	a	44,396	1,411	80	31	28	396	2.40	26	1,334	21	32	412
P. octaurelia	K8	72.98	28.68	a	38,668	1,471	82	40	41	401	2.51	26	1,381	24	37	362
P. pentaurelia	87	87.30	23.18	a	41,676	1,482	83	27	20	397	2.56	27	1,412	2	3	547
P. primaurelia	AZ9–3	86.33	23.02	a	42,615	1,449	82	22	19	400	2.46	26	1,375	18	29	533
P. quadecaurelia	N1A	59.12	23.21	a	33,793	1,388	79	44	40	399	2.33	26	1,303	25	34	351
P. sexaurelia	AZ8–4	68.02	24.07	a	36,094	1,432	81	18	33	400	2.43	26	1,362	10	16	433
P. sexaurelia	CA1	68.02	24.07	b	-	-	-	-	-	-	-	-	-	-	-	-
P. sonneborni	30995	98.06	23.02	a	49,951	1,384	79	32	26	399	2.31	26	1,306	24	33	539
P. tetraurelia	51	72.10	28.04	a	40,460	1,425	80	55	69	408	2.35	25	1,329	23	36	328

P. tetraurelia	d4–2	72.09	28.05	a	39,642	1,429	80	5	19	418	2.28	25	1,359	64	52	385
*P. tetraurelia**	51	98.49	27.35	a	-	-	-	-	-	-	-	-	-	-	-	-

A, species; B, strain name; C, genome size (in Mbp); D, GC%; E, data sources: a-ParameciumDB, b-NCBI: GCA_000733375.1; F, number of protein-coding genes; G, gene length (in bp); H, proportion of genes with introns in percentage; I, proportion of genes with five-prime UTRs in percentage; J, proportion of genes with three-prime UTRs in percentage; K, mean exon length (in bp); L, mean number of introns per gene; M, mean intron length (in bp); N, mean length of CDSs (in bp); O, mean length of five-prime UTRs (in bp); P, mean length of three-prime UTRs (in bp); Q, mean intergenic length (in bp); *, micronuclear genome.

Supplemental Table 2 Genomic information on non-*aurelia Paramecium* species

A	B	C	D	E	F	G	H	I	J	K	L	M	N	O	P	Q	R
P. bursaria	110224	29.16	28.75	a	-	-	-	-	-	-	-	-	-	-	-	-	-
P. bursaria	Dd1	26.82	28.79	b	15,591	1,437	85	41*	41*	3.46	396	2.46	28	1,347	33	20	203
P. bursaria	HK1	26.82	28.80	c	15,414	1,450	85	40*	41*	3.43	403	2.43	27	1,360	34	21	203
P. bursaria	KM2	26.82	28.80	d	15,278	1,461	85	39*	41*	3.44	405	2.44	27	1,371	33	21	205
P. bursaria	STL3	26.82	28.84	e	15,280	1,453	84	39*	40*	3.40	409	2.40	27	1,366	33	21	209
P. caudatum	43c3d	30.53	28.20	a	8,713	1,462	82	19	44	3.40	413	2.40	23	1,384	27	36	2,010
P. multimicro-nucleatum	MO3c4	35.73	25.26	a	-	-	-	-	-	-	-	-	-	-	-	-	-

A, species; B, strain name; C, genome size (in Mbp); D, GC%; E, data sources: a-ParmeciumDB, b-NCBI: GCA_016759035.1, c-GCA_016808045.1, d-GCA_016808065.2, e-GCA_016802775.1; F, number of protein-coding genes; G, gene size (in bp); H, proportion of genes with introns in percentage; I, proportion of genes with five-prime UTRs in percentage; J, proportion of genes with three-prime UTRs in percentage; K, mean exon number per gene; L, mean exon length (in bp); M, mean number of introns per gene; N, mean intron length (in bp); O, mean length of CDSs (in bp); P, mean length of five-prime UTRs (in bp); Q, mean length of three-prime UTRs (in bp); R, mean intergenic length (in bp). * Probably resulting from the annotations in lack of Cap-Seq data, the majority of UTRs start and end at the same genome coordinate and are here removed.
